# Natural progress of D-dimer following total joint arthroplasty: a baseline for the diagnosis of the early postoperative infection

**DOI:** 10.1186/s13018-018-0730-4

**Published:** 2018-02-13

**Authors:** Yong Seuk Lee, Young-Kyun Lee, Seung Bum Han, Chang Hyun Nam, Javad Parvizi, Kyung-Hoi Koo

**Affiliations:** 1 Department of Orthopedic Surgery, Seoul National University College of Medicine, Seoul National University Bundang Hospital, 82 Gumi-ro, 173 Beon-gil, Bundang-gu, Seongnam, Gyeonggi-do 463-707 South Korea; 2 Department of Orthopedic Surgery, Korea University Anam Hospital, Seoul, South Korea; 3 Department of Orthopedic Surgery, Mokdong Himchan Hospital, Seoul, South Korea; 4 Rothman Institute at Thomas Jefferson University, Philadelphia, PA USA

**Keywords:** D-Dimer, Periprosthetic joint infection, Total hip arthroplasty, Total knee arthroplasty

## Abstract

**Background:**

Early detection followed by prompt intervention is essential for the treatment of periprosthetic joint infection (PJI). D-dimer, a fibrin degradation product, characteristically changes rapidly during early postoperative period and has a short half-life. The aim of this prospective study was to measure postoperative change of D-dimer level after joint arthroplasty in conjunction with ESR and CRP.

**Methods:**

ESR, CRP, and D-dimer levels were measured on the day before surgery, postoperative days 1, 2, 3, and 5 and weeks 2 and 6 in 65 patients who underwent elective primary total hip arthroplasty (38 hips in 38 patients) or total knee arthroplasty (27 knees in 27 patients). We compared perioperative changes of the three biomarkers.

**Results:**

ESR level was elevated from postoperative day 1 and reached a peak level of 45 mm/h at postoperative day 5. The elevation persisted until postoperative week 6. CRP level was elevated from postoperative day 1 and reached a peak level of 10 mg/dl between postoperative day 2 and day 3. The CRP level was decreased to the normal level around postoperative week 2. D-dimer level was sharply elevated and peaked to 4.5 μg/dl at postoperative day 1. At postoperative day 2, it decreased to baseline level. After then, it slowly elevated again and reached a second peak at postoperative week 2.

**Conclusion:**

D-dimer showed a more rapid rise and fall than ESR and CRP in very early postoperative period. The D-dimer test might be effective in early detection of PJI, if combined with levels of ESR and CRP. The postoperative change of D-dimer in our study can serve as a baseline for early diagnosis of PJI.

## Background

Periprosthetic joint infection (PJI) after total hip arthroplasty (THA) or total knee arthroplasty (TKA) is one of the most dreadful complications and it has extremely negative effects on the physical, emotional, social, and economic aspects of a patient’s life [1–4]. To eradicate the infection, a diagnosis of PJI in its early stage is very important [4–7]. Serum erythrocyte sedimentation rate (ESR) and C-reactive protein (CRP) have been generally used as a screening test for infection because of their simplicity and cost-effectiveness [8]. However, they have low sensitivity and specificity and these may increase under several conditions in addition to infection [8–11]. Therefore, there have been some trials to establish a new single reference standard [6, 9].

D-dimers are fibrin degradation products formed as a result of fibrin clot dissolution by plasmin. This test is typically done in patients with suspected venous thromboembolism (VTE), manifested as deep vein thrombosis (DVT) or pulmonary embolism (PE). However, the increased fibrin production, which is manifested by the elevated D-dimer concentration, can also be observed in other clinical conditions, such as cancer, infection, inflammation, surgery, injuries, hemorrhages, and many others [12]. It could imply that elevated D-dimer level does not have a confirmatory value due to low specificity of the assay. However, it characteristically shows sharp increase up to double levels and decrease to the baseline level within a short time during early postoperative period. After then, it also shows second peak after maintaining at the steady level for a while [12]. With these characteristics, D-dimer was tried for the diagnosis of PJI. Serum D-dimer outperformed both ESR and serum CRP, with a sensitivity of 89% and a specificity of 93%. ESR and CRP had a sensitivity of 73 and 79% and a specificity of 78 and 80%, respectively [13].

Some patients could be suspected to have an infection at the immediate postoperative period. In this situation, rapid decision whether irrigation and debridement should be done to preserve the implanted prosthesis is an important issue because it is a time-honored procedure and it should be performed before the establishment of drug-resistant biofilm on the implant or before osteomyelitis becoming entrenched in periprosthetic bone [14]. However, CRP hits a peak on day 2 or 3 after surgery and diminishes to a normal value by postoperative week 2 and ESR shows slower natural progress than CRP. Therefore, we can miss the chance to save the implant using an early intervention if we only rely on these tests.

Thus, we were interested in whether D-dimer level shows a sharp change at an early postoperative period and it can be used as a promising biomarker for an early diagnosis of the infection by combining the natural progress with commonly used tests such as ESR and CRP. We hypothesized that the D-dimer will show a rapid change of the natural progress at a very early postoperative period. The aim of this study was to measure postoperative change of D-dimer level after joint arthroplasty in conjunction with ESR and CRP.

## Methods

Ethical approval for this prospective study was obtained from IRB of our hospital. Sixty-five volunteers scheduled for either elective primary THR (38 hips in 38 patients) or TKR (27 knees in 27 patients) surgery were recruited with their informed consent. Inclusion criteria are as follows: patients scheduled for THA or TKA due to primary or secondary osteoarthritis, not having significant preoperative comorbidity with a Charlson comorbidity index (CCI) < 2, and no sign of inflammation and adverse event during test period. Exclusion criteria are as follows: (1) inflammatory arthritis such as rheumatoid arthritis, (2) patients who have taken preoperative pharmacologic DVT prophylaxis or suspicious DVT, (3) revision THA or TKA, and (4) patients who have missing data at all scheduled time points.

All TKAs were performed with an intraoperative tourniquet. The tourniquet was inflated just before the skin incision and deflated after the cementation was completed. After a midline skin incision, a medial parapatellar approach was performed. Intramedullary guide was used for the femur and extramedullary guide for the tibia. The posterior cruciate was sacrificed and fixed-bearing TKA were implanted in all patients. All prostheses were fixed with cement. An intra-articular closed-suction drainage after TKA was used for 24 h. All THAs were performed with the Kocher-Langenbeck approach. The short external rotators were detached with electrocautery as close as possible from their insertion, and posterior capsule was exposed. After posterior capsulotomy, the femoral head was dislocated posteriorly. The femoral neck was cut at the base and the femoral head was removed. The femoral stem and acetabular cup were inserted through the posterior approach without cement. After implantation of the prosthesis, we repaired the capsule and short external rotators. Closed-suction drainage after THA was also used for 24 h. We used tranexamic acid at subcutaneous tissue both THA and TKA.

ESR, CRP, and D-dimer values were collected on the day before surgery, postoperative days 1, 2, 3, and 5 and weeks 2 and 6. The D-dimer level in venous plasma was assessed by an immunoturbidimetric assay, STA Liatest D-Di (Diagnostica Stago, Asnieres sur Seine, France) on a STA-R analyzer (Diagnostica Stago). According to the data provided by the manufacturer (Diagnostica Stago), the measuring range of STA Liatest is from 0.27 μg/ml (fibrinogen equivalent unit, FEU) to 4.0 μg/ml without dilution and with dilution up to 20.0 μg/ml (FEU). The cutoff value of 0.50 μg/ml showed a negative predictive value of 95 to 100% from two studies that tested the use of STA Liatest D-Di as an aid in the diagnosis of DVT [15, 16].

For the evaluation, preoperative demographic data and values of each test at the scheduled time points were compared between THA and TKA. In addition, change of the percentage of each test value compared to the maximal level at the early postoperative period was also performed to verify the effectiveness of each test.

### Statistical analysis

Statistical analysis was performed using R version 3.3.1 (The R Foundation for Statistical Computing). Comparison of ESR, CRP, and D-dimer between preoperative and postoperative values was performed using a paired *t* test because most of variables followed normal distribution. Comparison on the early postoperative value of the D-dimer was performed using Tukey HSD test.

### Funding source

This study was supported by Corentec (Cheonan, South Korea).

## Results

**Fig. 1:**
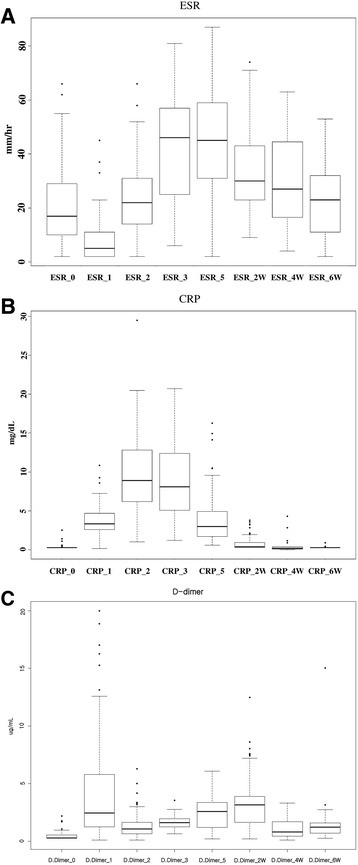
Values of each test (**a** ESR, **b** CRP, and **c** D-dimer) that are presented as a box plot at each time points; median line in box plot means median, and box means quartile range (QR)

Detailed demographic data of patients were presented in Table 1. There was a male dominance in THA patients (M:F = 24:14) and female dominance in TKA patients (M:F = 3:24). Age was older in TKA group than in THA group. Values of each test at the scheduled time points were presented as a box plot in Fig. 1. Mean and standard deviation (SD) values were presented and these were compared between THA and TKA in Table 2. In ESR, values showed a statistically significant difference between groups at the postoperative day 2 and week 2 and they were not consistently high or low in each group. In CRP, postoperative 2-week value showed a statistically significant difference between groups and higher value was observed in the TKA group. In D-dimer, values showed a statistically significant difference between groups at the preoperative value and postoperative day 5 and they were consistently high in the THA group.

**Table 1:** Demographic data

	THA			TKA		
Dx	AVN	OA	*p*	OA	AVN	*p*
	(*N* = 19)	(*N* = 19)		(*N* = 25)	(*N* = 2)	
Sex						
F	1 (5.3%)	13 (68.4%)		23 (92.0%)	1 (50.0%)	
M	18 (94.7%)	6 (31.6%)		2 (8.0%)	1 (50.0%)	
Age	54.6 ± 14.2	60.1 ± 10.9	0.192	69.9 ± 7.1	76.5 ± 0.7	0.207
Weight	65.6 ± 10.6	64.3 ± 7.1	0.654	61.2 ± 9.9	67.0 ± 8.5	0.431
Height	169.8 ± 6.7	158.7 ± 9.7	0	151.7 ± 7.7	157.5 ± 0.7	0.306
BMI	22.7 ± 3.4	25.7 ± 3.2	0.01	26.7 ± 4.8	27.0 ± 3.7	0.924

**Table 2:** Values of each test at the scheduled time points

POD	THA	TKA	*p* value	POD	THA	TKA	*p* value	POD	THA	TKA	*p* value
	(*N* = 38)	(*N* = 27)			(*N* = 38)	(*N* = 27)			(*N* = 38)	(*N* = 27)	
ESR_0	21.4 ± 17.4	19.6 ± 12.0	0.636	CRP_0	0.4 ± 0.4	0.3 ± 0.1	0.073	D-dimer_0	0.6 ± 0.4	0.4 ± 0.3	0.019
ESR_1	9.6 ± 10.3	7.3 ± 7.9	0.328	CRP_1	4.0 ± 2.1	3.7 ± 1.8	0.558	D-dimer_1	5.2 ± 4.9	3.9 ± 5.8	0.351
ESR_2	27.9 ± 15.5	18.0 ± 12.8	0.008	CRP_2	9.5 ± 4.4	10.2 ± 5.9	0.573	D-dimer_2	1.5 ± 1.0	1.3 ± 1.4	0.607
ESR_3	44.3 ± 18.9	35.1 ± 22.1	0.212	CRP_3	8.6 ± 4.4	10.5 ± 6.0	0.306	D-dimer_3	1.6 ± 0.5	1.9 ± 0.8	0.157
ESR_5	45.8 ± 19.6	44.3 ± 18.5	0.749	CRP_5	4.2 ± 3.0	4.2 ± 4.2	0.943	D-dimer_5	3.1 ± 1.2	1.7 ± 1.4	0
ESR_2W	30.6 ± 13.8	40.0 ± 18.3	0.022	CRP_2W	0.5 ± 0.6	1.2 ± 1.1	0.005	D-dimer_2W	3.7 ± 1.7	2.7 ± 3.0	0.13
ESR_6W	22.0 ± 14.7	28.4 ± 12.1	0.228	CRP_6W	0.3 ± 0.1	0.3 ± 0.0	0.125	D-dimer_6W	1.5 ± 2.4	1.8 ± 0.5	0.502

**Fig. 2:**
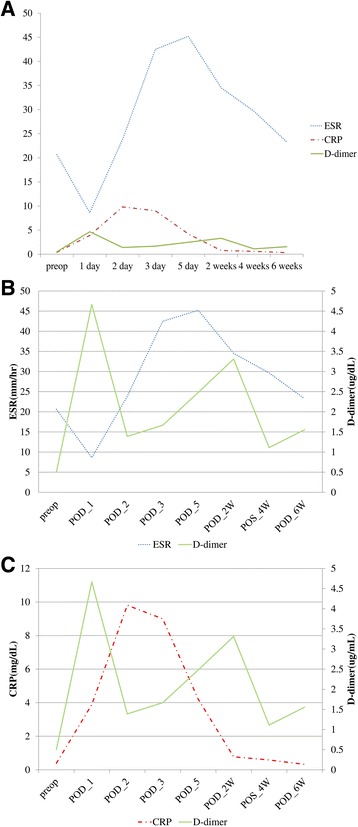
Serial change of each test that is presented as a graph (**a**); comparative graphs were also presented between ESR and D-dimer (**b**) and between CRP and D-dimer (**c**)

Serial change of each test was diagramed in Fig. 2. In Fig. 2a, all tests were presented using a serial graph. Comparative graphs between D-dimer and ESR and between D-dimer and CRP were presented in Fig. 2b, c. ESR elevated from postoperative day 1, peaked at postoperative day 5 (peak level 45 mm/h), and was still high at the postoperative week 6. CRP elevated from postoperative day 1, peaked between postoperative days 2 and 3 (peak level 10 mg/dl), and decreased to the normal level at around postoperative week 2. D-dimer sharply elevated and peaked at postoperative day 1 (peak level 4.5 μg/dl). At postoperative day 2, it decreased to nearly baseline level. After then, it slowly elevated again and reached second peak at postoperative week 2.

**Fig. 3:**
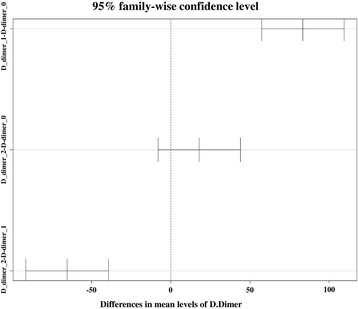
Comparison of the D-dimer level at the early postoperative period; differences in mean levels were presented as 95% confidence interval

Change of the percentage of each value compared to the maximal level at the early postoperative period was presented in Table 3. Elevation of the D-dimer during postoperative day 1 showed a statistically significant difference (83.4% elevation compared to the maximal level, *p* = 0.000) and decrease during postoperative day 2 also showed a statistically significant difference (65.5% decrease compared to the maximal level, *p* < 0.001). However, the change between postoperative days 2 and 3 was not statistically significant (2.2% elevation compared to the maximal level, *p* = 0.303). ESR and CRP also showed statistically significant differences during postoperative days 1, 2, and 3, but changes were smaller compared to those of D-dimer. In the comparison of the D-dimer level, they showed statistically significant differences between baseline and postoperative day 1 and between postoperative days 1 and 2. However, there was no statistical difference between baseline and postoperative day 2 and the level of the D-dimer decreased to the baseline level at the postoperative day 2 (Fig. 3).

**Table 3:** Change of the percentage of each test value compared to the maximal level at the early postoperative period

	PreOP vs POD_1	POD_1 vs POD_2	POD_2 vs POD_3
D-dimer			
Mean difference (%)	83.4	− 65.5	2.2
*p* value	0.000	0.000	0.303
CRP			
Mean difference (%)	34.9	59.2	− 5.0
*p* value	0.000	0.000	0.034
ESR			
Mean difference (%)	− 24.1	30.4	29.9
*p* value	0.000	0.000	0.000

## Discussion

The principal findings of this study were (1) ESR started to increase at postoperative day 1, hits a peak on day 5, and maintained a slightly elevated level at postoperative week 6; (2) CRP started to increase after operation, hits a peak on day 2 or 3 and diminished to a normal level at postoperative week 2; and (3) D-dimer sharply increased after operation, hits a peak on day 1, sharply decreased on day 2, after then slowly increased up to postoperative week 2, and decreased to the nearly normal level at postoperative week 6. From our analysis, we could assume that absence of the sharp decrease on day 2 or 3 in D-dimer may be abnormal. Additionally, it was also assumed that it would be abnormal if there is no decrease on day 5 results of the ESR and CRP compared to results of day 3 and an additional sharp increase of the D-dimer.

Lower limb orthopedic surgery such as THA and TKA results in increased coagulation and inflammatory reactions postoperatively [17]. Oelsner et al. [18] reported that acute phase response was both predictable and consistent in healthy person after THA and TKA; however, there was a different response to TKA and THA and the peak CRP and fibrinogen were significantly higher for TKA than THA. In a study by Hughes et al. [17], THA and TKA resulted in similar changes of coagulation and non-specific inflammatory biomarkers such as ESR, CRP, and D-dimer. Bytniewski et al. [12] also reported that the distribution of D-dimer values throughout the entire postoperative period was not specific in any group.

Most important problem of biomarkers is that they are not specific to the infection and they can be elevated in with various causes. Kim et al. [8] evaluated the causes of elevated CRP level in the early postoperative period after primary TKA. They reported that 24% caused by postoperative infection, 20% by idiopathic cause, 16% by urologic problem, 14% by gastrointestinal problem, 13% by vascular problem, and 13% by respiratory problem. Therefore, diagnosis of PJI commonly relies on a combination of clinical judgment, serologic testing, information obtained from joint aspiration, radiologic assessment, and microbiologic as well as histopathologic testing obtained at the surgery [19, 20]. However, of these methods, serologic tests are most widely used as a primary screening test to predict infection.

D-dimer was originally used in screening DVT or PE after total joint arthroplasty. It has been also controversial in diagnosing DVT or PE because of low specificity [12, 21–23]. However, one important characteristic of D-dimer is that it shows sharp increase up to double levels and decrease to the baseline level within a short time during early postoperative period [12]. According to recent literature, serum D-dimer is a marker for the diagnosis of PJI, and the median D-dimer level was significantly higher for the patients with PJI than for the patients with aseptic failure [13]. Our results also showed the sharp increase and decrease within postoperative day 1, and it hit the peak more than double levels within a short time period. Additionally, it showed dull slope after the abrupt change even though there was second peak at postoperative week 2. It means that it only takes within 1 day for the first peak and more than 2 weeks for the second peak. Therefore, this information could be helpful in diagnosing postoperative infection in the earlier time period with combination of basic screening tests such as ESR and CRP.

Our study has some limitations that should be considered. First, there was no control group and serial assessment was only performed with patients who were not infected. Therefore, we do not know how values can change after infection. Second, time interval between postoperative day 5 and week 2 and between weeks 2 and 6 is long; there could be different results in terms of peak level and period of the ESR and second peak of the D-dimer. Third, we usually perform diagnostic procedure for detecting DVT in patients who show symptoms. However, there was no patient who showed suspicious symptoms of DVT. Therefore, we cannot know how many patients had silent DVT or pulmonary thromboembolism. Finally, values showed a little large range. Therefore, it would be a little difficult to apply all patients that have different characteristics. Nevertheless, we could find a dramatic change of the D-dimer level during early postoperative period and it could enable us to determine whether the arthroplasty is abnormal or not. Furthermore, the judgment would be more accurate if we consider all three tests together.

## Conclusion

D-dimer showed a rapid and different pattern of the change at a very early postoperative period. It was assumed that this test would be used effectively in diagnosing early postoperative infection with the combination of the ESR and CRP. This natural progress can serve as a baseline for early diagnosis of infection.

## Abbreviations

CCI: Charlson comorbidity index
CRP: C-reactive protein
DVT: Deep vein thrombosis
ESR: Erythrocyte sedimentation rate
PE: Pulmonary embolism
PJI: Periprosthetic joint infection
SD: Standard deviation
THA: Total hip arthroplasty
TKA: Total knee arthroplasty
VTE: Venous thromboembolism
